# Prognostication of Brain-Metastasized Patients Receiving Subsequent Systemic Therapy: A Single-Center Long-Term Follow-Up

**DOI:** 10.3390/curroncol32020074

**Published:** 2025-01-28

**Authors:** Tijl Vermassen, Charlotte Van Parijs, Stijn De Keukeleire, Katrien Vandecasteele, Sylvie Rottey

**Affiliations:** 1 Department Medical Oncology, Ghent University Hospital, 9000 Ghent, Belgium; 2 Biomarkers in Cancer, Department Basic and Applied Medicine, Ghent University, 9000 Ghent, Belgium; 3 Cancer Research Institute Ghent, 9000 Ghent, Belgium; 4 Department Radiation Oncology, Ghent University Hospital, 9000 Ghent, Belgium; 5 Radiotherapy and Experimental Cancer Research, Department Human Structure and Repair, Ghent University, 9000 Ghent, Belgium; 6 Drug Research Unit Ghent, Ghent University Hospital, 9000 Ghent, Belgium

**Keywords:** brain metastases, targeted therapy, survival outcome, prognosis, retrospective analysis

## Abstract

Background. Survival of patients with brain metastases (BMs) is poor. It has become clear that targeted therapy has an effect on BMs and patient’ prognosis. The question remains which patients benefit from additional systemic therapy. This assumption was evaluated in a large single-center cohort. Methods. Patients consecutively planned to undergo local radiotherapy for their BMs in 2006–2017 were selected (*n* = 200). Prognosis, using CERENAL, disease-specific graded prognostic assessment (DS-GPA), and Radiation Therapy Oncology Group recursive partitioning analysis (RTOG RPA), was evaluated. Results. Ninety-three (46.5%) patients received at least one additional line of systemic therapy subsequent to the diagnosis of their BMs. The median overall survival (OS) was 6.3 months. Having received subsequent systemic therapy resulted in a more favorable OS (10.4 versus 3.9 months). Interestingly, using dichotomized scores, CERENAL showed prognostic properties in all patients for disease-specific survival on multivariate analysis, whereas RTOG RPA and DS-GPA were not withheld in the model. Lastly, only having a favorable DS-GPA resulted in prolonged progression-free survival for first systemic therapy following BM diagnosis. Conclusions. Receiving subsequent systemic therapy has a profound influence on outcome in patients with BMs, indicating the effect of systemic therapy on BMs. Use of the CERENAL brain prognostic score shows potential for further prognostication of patients with more favorable outcomes.

## 1. Introduction

Brain metastases (BMs) are frequently observed in patients with metastatic solid tumors. Recent reports indicate incidence rates up to 45% due to an aging population, novel local and systemic therapies, and improved diagnostic techniques for BMs, such as magnetic resonance imaging [1]. The most frequently BM-associated primary malignancies are lung cancer (16.3%), renal cell carcinoma (RCC; 9.8%) melanoma (7.4%) and breast cancer (5.0%) [1]. Symptomatic BMs can lead to fatigue, headache, seizures, focal weakness or numbness, and cognitive impairments [2,3,4].

A first therapeutic option is surgical resection for bulky and symptomatic BMs that are not present in an eloquent area. Important advantages are the possibility of histological diagnosis, reduction in neurological deficits and seizures, and an immediate relief of symptoms of intracranial hypertension [5,6]. Otherwise, radiotherapy is frequently used to treat patients with BMs. Depending on the number of brain lesions present (oligometastatic disease), the preferred choices are stereotactic radiosurgery (SRS) and whole-brain radiation therapy (WBRT). The former has shown to have improved cognitive outcomes over WBRT, whereas the advantage of WBRT is the targeting of possible hematogeneous disseminated micrometastases [5,6]. Nowadays, systemic therapies have become of interest to treat BMs. Due to advances in therapeutic development and newer, smaller, and more lipophilic molecules, targeted therapies can cross the blood–brain barrier and target BMs. In this regard, EGFR and ALK tyrosine kinase inhibitors, e.g., osimertinib and brigatinib have been tailored based on their observed activity against BMs [6,7,8,9,10].

Nevertheless, despite various treatment modalities, survival remains poor, with median overall survival (OS) of 12 months [11,12]. Recently, the American Society for Radiation Oncology updated their practice guidelines. They stated that the management of patients with BMs depends on the number of BMs, the ECOG performance status, and estimated prognosis, emphasizing the importance of prognostic factors [6].

Several BM prognostic factors have been developed. One validated score is the Radiation Therapy Oncology Group recursive partitioning analysis (RTOG RPA), including four factors positively associated with OS: Karnofsky performance status (KPS) > 60, controlled primary tumor, age < 60 years old, and no extracranial metastases. As RTOG RPA is limited by varying underlying clinical factors, more complex models were constructed, such as disease-specific graded prognostic assessment (DS-GPA), which take the specific hallmarks of individual primary tumor types into account [13,14,15]. The advantage of DS-GPA is that these nomograms have been modified to specific solid malignancies. In addition, DS-GPA is continuously updated allowing for the incorporation of novel covariates, such as mutational status, thus taking the changing therapeutic landscape into account in its prognostic properties [16,17,18,19,20]. However, to our knowledge, DS-GPA has not specifically been evaluated as a prognostic parameter for progression-free survival (PFS) in patients who receive subsequent systemic therapy following diagnosis of their BMs. Due to the increasing importance of systemic therapy to manage BMs, our group has retrospectively highlighted the prognostic properties of a generalized, more extensive brain metastatic prognostic score (CERENAL) in brain-metastasized RCC patients treated with targeted agents [21].

The question remains if the CERENAL score enables stratification of patients with BMs from various solid carcinomas to having a poor or favorable prognosis depending on whether or not they have received additional systemic therapy following diagnosis of their BMs. Simultaneously, it needs to be confirmed if brain prognostic scores enable prediction of which patients will have a longer PFS during first subsequent systemic therapy following BM diagnosis. We therefore retrospectively evaluated a modified CERENAL score, next to the validated and widely used RTOG RPA and DS-GPA, in a large single-center cohort of patients with BMs planned to receive primary local radiotherapy for their BMs.

## 2. Materials and Methods

### 2.1. Patient Selection

In this single-center retrospective analysis, 208 patients with a solid carcinoma consecutively planned to receive local radiotherapy for their BMs at Ghent University Hospital in the period January 2006 and December 2017 were included. Eight patients were excluded due to presence of a primary brain tumor (*n* = 4), no confirmed diagnosis of BMs (*n* = 2), or no data available in the electronic patient files (*n* = 2). The STARD flow diagram is given in Figure S1. Patient data were retrieved from the electronic patient files. The study was approved by the Ethics Committee of Ghent University Hospital (Belgian registration: B670201837399).

### 2.2. Brain Prognostic Factors

BM prognostic scores were determined as described in the literature. Patients were distributed among RTOG RPA classes I, II, or III using general health risk factors such as age, KPS, and presence of extracranial metastases at BM diagnosis [22]. Secondly, DS-GPA was calculated through the sum of the separate prognostic factors included in the tumor subtype-specific nomogram, as indicated in the literature [16,17,18,19,20]. In contrast to RTOG-RPA, DS-GPA contains both general health risk factors and disease-specific risk factors, such as f.i. mutational status. Lastly, CERENAL was calculated by attributing a point per poor prognostic factor. Comparable to RTOG-RPA, CERENAL is only composed of general health risk factors. Next to KPS, age at BM diagnosis, and other extracranial metastases, we also included some other relevant risk factors, such as systemic disease, number of brain metastatic lesions, and no boost, SRS, or neurosurgery received. Calculation of each prognostic score is given in Table 1.

Due to the more heterogeneous cohort in the current research, some of the cut-off values for prognostic factors used in the previously described CERENAL prognosticator no longer resulted in or resulted in less significant outcomes [21]. Therefore, the CERENAL score was adapted ad hoc by means of ROC curve analysis to determine the most appropriate cut-off value (highest Youden index). As a result, the modified covariates were more suitable for the patient characteristics in this expanded and more heterogeneous cohort. As none of the abovementioned prognostic scores had included neurological symptoms as potential covariates, the prognostic effect of presence/absence of neurological symptoms was evaluated.

### 2.3. Statistical Analysis

Prognostic scores were analyzed as multicategorical and dichotomized variables (based on median values). Spearman’s rank correlation coefficient ρ and Fisher’s exact or chi-squared tests assessed the association between multicategorical variables and dichotomized prognostic scores, respectively. Difference in time interval between BM diagnosis and subsequent systemic treatment initiation for prognostic scores and primary local therapies was determined using the Kruskal–Wallis test. Progression-free survival (PFS; time from initiation of subsequent systemic treatment after BM diagnosis until radiographic progression or death, whichever occurred first), OS (time from BM diagnosis until death or last follow-up), and disease-specific survival (DSS; time from BM diagnosis until disease-specific death or last follow-up) were calculated for each BM prognostic score (multicategorical and dichotomized) using a 2-sided Mantel–Cox log-rank test. Patients that were lost to follow-up were censored in the survival analysis. Survival curves were plotted using the Kaplan–Meier method. The covariate effects of the statistically significant scores were determined in a multivariate analysis via the Cox proportional hazard model (backward method). *p* < 0.05 was considered statistically significant. Analyses were performed with MedCalc Statistical Software v20.110 (MedCalc Software, Ostend, Belgium); and GraphPad Prism v8.0.2 (GraphPad Software Inc., La Jolla, CA, USA).

## 3. Results

### 3.1. Patient Characteristics

An overview of all clinical characteristics at diagnosis of the primary tumor in the intention-to-treat (ITT) cohort is given in Table S1. Most notably, gender was evenly distributed. A majority of patients were diagnosed with lung cancer (38%, of which 72% were a non-small-cell lung adenocarcinoma). Eighty-three patients (41.5%) in the ITT cohort were diagnosed with T3/T4 tumors. Extensive disease was frequently seen at primary diagnosis in the ITT cohort, with 58% and 54% of patients having T3/T4 tumors, lymph node involvement or metastatic disease, respectively.

Median age at BM diagnosis was 62 years, with a median time from primary tumor diagnosis to BM diagnosis of 15 months. Overall, 27% of patients presented with BMs at time of primary tumor diagnosis and 84.5% of brain-metastasized patients had concurrent extracranial metastases. A total of 109 patients (54.5%) presented with three or more brain lesions at BM diagnosis. Neurological symptoms were noted in 78% of patients. Almost every patient (96.5%) received primary local radiotherapy for treatment of their BMs. In addition, 27 patients underwent additional primary neurosurgery. Patients receiving neurosurgery had a median of one BM, whereas patients receiving no neurosurgery had a median of three BMs (*p* < 0.0001). In sum, 93 patients (46.5%) and 73 patients (36.5%) received at least one additional line of systemic therapy subsequent to BM diagnosis and subsequent to primary local therapy for BMs, respectively. An overview of clinical characteristics at BM diagnosis and therapies administered is given in Table S2.

### 3.2. Brain Prognostic Scores

Patient distribution among RTOG RPA, DS-GPA, and CERENAL is given in Table 2.

Despite a significant association, the concordance between RTOG RPA and CERENAL was only 57%, with a discrepancy seen for distribution of patients with RTOG RPA I/II among CERENAL 0–3 (48%) and CERENAL 4–6 (52%) scores. In addition, only a moderate concordance was observed between RTOG RPA and DS-GPA. The highest agreement was between DS-GPA and CERENAL (ρ = −0.681). Nevertheless, patients with a CERNAL score 0–3 were equally distributed among DS-GPA 0.0–2.0 and 2.5–4.0 (n = 39 in each group). Next, the type of primary local therapy administered for BM was highly associated with CERENAL and to a lesser extent with RTOG-RPA and DS-GPA. Interestingly, RTOG RPA was associated with whether or not subsequent systemic therapy was received (*p* = 0.0025), whereas DS-GPA (*p* = 0.9349) and CERENAL were not (*p* = 0.4286).

### 3.3. Survival Outcome

**Figure 1:**
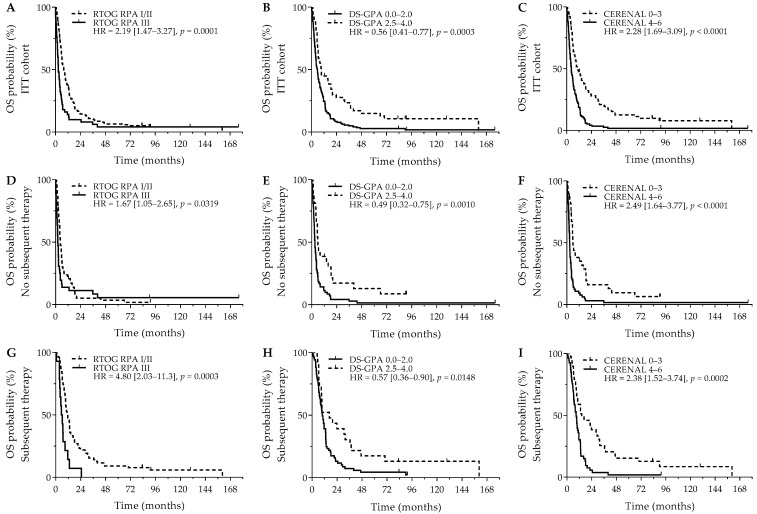
OS outcomes for brain metastasis prognostic scores. *Y*-axis depicts cumulative survival (%), *X*-axis depicts survival time in months. OS curves are shown for: (**A**) RTOG RPA in the ITT cohort (*p* = 0.0001), (**B**) DS-GPA in the ITT cohort (*p* = 0.0003), (**C**) CERENAL in the ITT cohort (*p* < 0.0001), (**D**) RTOG RPA in patients who did not receive subsequent systemic therapy (*p* = 0.0319), (**E**) DS-GPA in patients who did not receive subsequent systemic therapy (*p* = 0.0010), (**F**) CERENAL in patients who did not receive subsequent systemic therapy (*p* < 0.0001), (**G**) RTOG RPA in patients who received subsequent systemic therapy (*p* = 0.0003), (**H**) DS-GPA in patients who received subsequent systemic therapy (*p* = 0.0148), and (**I**) CERENAL in patients who received subsequent systemic therapy (*p* = 0.0002). HRs (95% CIs) are indicated in each survival graph. CI, confidence interval; DS-GPA, diagnosis-specific graded prognostic assessment; HR, hazard ratio; OS, overall survival; RTOG RPA, Radiation Therapy Oncology Group recursive partitioning analysis.

After a median follow-up of 7.5 years, the median OS was 6.3 months (95%CI = 5.4 months–8.0 months). Patients receiving subsequent systemic therapy after diagnosis of their BMs had a significantly better OS outcome versus patients who did not receive subsequent systemic therapy (10.4 versus 3.9 months; *p* < 0.0001). Interestingly, having neurological symptoms was not related to a significant worse OS outcome (5.9 months versus 9.1 months; HR = 1.35, 95% Cl (0.97–1.88); *p* = 0.0785). Next, RTOG RPA (*p* = 0.0002), DS-GPA (*p* = 0.0001), and CERENAL (*p* < 0.0001) were significantly associated with OS. Using dichotomized scores, RTOG RPA I/II (8.1 versus 2.9 months; *p* = 0.0001), DS-GPA 2.5–4.0 (9.4 versus 5.7 months; *p* = 0.0003), and CERENAL 0–3 (10.9 versus 4.5 months; *p* < 0.0001) resulted in an improved survival outcome, with CERENAL and RTOG RPA being the only independent prognosticators for OS (Figure 1A–C). All brain prognostic scores proved to be significant in the subanalysis for patients that did not receive subsequent systemic therapy. Although favorable RTOG-RPA I/II (4.5 months versus 2.1 months; *p* = 0.0319), DS-GPA 2.5–4.0 (5.9 months versus 3.0 months; *p* = 0.0010), and CERENAL 0–3 (5.8 months versus 2.6 months; *p* < 0.0001) showed prognostic potential on univariate analysis, CERENAL was the only independent prognosticator in this patient cohort (*p* < 0.0001; Figure 1D–F). A second subanalysis for patients who received any subsequent therapy following BM diagnosis resulted in similar significant results for RTOG-RPA I/II (11.6 months versus 5.6 months; *p* = 0.0003), DS-GPA 2.5–4.0 (16.6 months versus 9.9 months; *p* = 0.0148), and CERENAL 0–3 (14.4 months versus 8.9 months; *p* = 0.0002; Figure 1G–I). In this patient cohort, however, both RTOG-RPA and CERENAL (*p* = 0.0330 and *p* = 0.0032, respectively) were withheld as independent prognosticators during multivariate analysis. All data regarding OS outcomes are given in Table 3 and Table S3.

**Figure 2:**
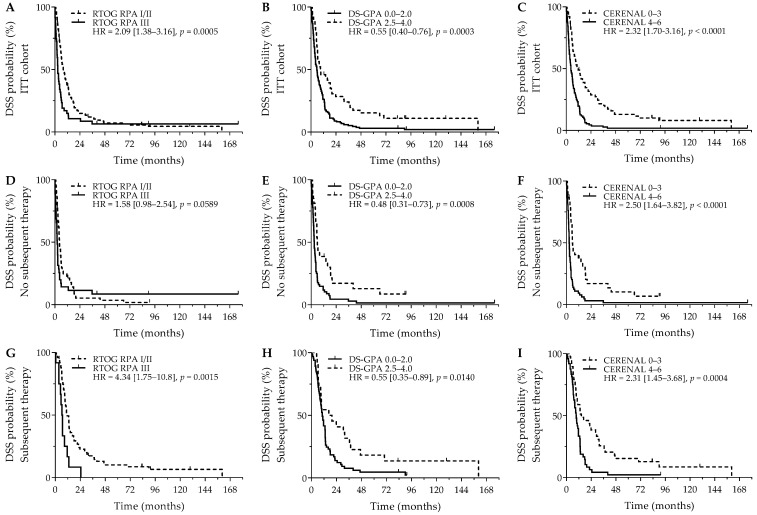
DSS outcome for brain metastasis prognostic scores. *Y*-axis depicts cumulative survival (%), *X*-axis depicts survival time in months. DSS curves are demonstrated for: (**A**) RTOG RPA in the ITT cohort (*p* = 0.0005), (**B**) DS-GPA in the ITT cohort (*p* = 0.0003), (**C**) CERENAL in the ITT cohort (*p* < 0.0001), (**D**) RTOG RPA in patients who did not receive subsequent systemic therapy (*p* = 0.0589), (**E**) DS-GPA in patients who did not receive subsequent systemic therapy (*p* = 0.0008), (**F**) CERENAL in patients who did not receive subsequent systemic therapy (*p* < 0.0001), (**G**) RTOG RPA in patients who received subsequent systemic therapy (*p* = 0.0015), (**H**) DS-GPA in patients who received subsequent systemic therapy (*p* = 0.0140), and (**I**) CERENAL in patients who received subsequent systemic therapy (*p* = 0.0004). HRs (95% CIs) are indicated in each survival graph. CI, confidence interval; DS-GPA, diagnosis-specific graded prognostic assessment; DSS, disease-specific survival; HR, hazard ratio; RTOG RPA, Radiation Therapy Oncology Group recursive partitioning analysis.

Comparable survival outcomes were obtained for evaluation of DSS. As was seen for OS, RTOG RPA I/II (8.1 versus 2.9 months; *p* = 0.0005), DS-GPA 2.5–4.0 (9.4 versus 5.8 months; *p* = 0.0003), and CERENAL 0–3 (10.7 versus 4.5 months; *p* < 0.0001) showed a favorable DSS outcome (Figure 2A–C). For DSS, however, only CERENAL was considered an independent prognosticator on multivariate analysis. In the cohort of patients who did not receive subsequent systemic therapy, RTOG-RPA I/II was only borderline significant (4.5 months versus 2.3 months; *p* = 0.0589), whereas DS-GPA 2.5–4.0 (5.9 months versus 3.0 months; *p* = 0.0008) and CERENAL 0–3 (5.8 months versus 2.6 months; *p* < 0.0001) were associated with prolonged DSS (Figure 2D–F), with CERENAL being the independent prognosticator on multivariate analysis (*p* < 0.0001). In the cohort of patients who received any subsequent therapy, all brain prognostic scores—RTOG-RPA I/II (11.6 months versus 6.4 months; *p* = 0.0015), DS-GPA 2.5–4.0 (16.6 months versus 10.4 months; *p* = 0.0140) and CERENAL 0–3 (14.4 months versus 9.4 months; *p* = 0.0004)—proved significant on univariate analysis (Figure 2G–I), with again only CERENAL being withheld in the multivariate analysis (*p* = 0.0006). All data regarding DSS outcomes are given in Table 4 and Table S3.

Lastly, PFS properties of the brain prognostic scores were evaluated in patients receiving systemic therapy (chemotherapy (42%), targeted therapy (26%), immunotherapy (21%)) subsequent to BM therapy (*n* = 93). Median time to subsequent systemic therapy was significantly different between patients not receiving primary local therapy or only receiving WBRT 20 Gy (1.0 month) versus patients who received WBRT 30 Gy alone, WBRT 30 Gy + boost/neurosurgery/SRS, or SRS alone (2.3 months, *p* < 0.0001). Moreover, median time to subsequent systemic therapy was significantly different between poor and favorable dichotomized BM prognostic scores (RTOG RPA: 0.6 months versus 1.4 months, *p* = 0.0351; DS-GPA: 1.1 month versus 2.0 months, *p* = 0.0200; CERENAL: 1.0 month versus 2.2 months, *p* < 0.0001). In patients who received subsequent systemic therapy, absence of neurological symptoms surprisingly resulted in a shorter, although insignificant, PFS (median PFS 3.2 months versus 4.2 months; HR = 1.44, 95% Cl (0.89–2.35); *p* = 0.1410). Next, RTOG RPA, DS-GPA, and CERENAL were significantly associated with PFS (Table S3). However, further dichotomization resulted in a significant PFS outcome for DS-GPA (*p* = 0.0360), but not for RTOG RPA (*p* = 0.3163) and CERENAL (*p* = 0.0506; Figure S2A–C). Interestingly, using intracranial PD as end point resulted in a longer, though insignificant prolonged PFS for patients with a favorable score for DS-GPA (9.4 months versus 4.9 months; HR_DS-GPA 2.5–4.0_ = 0.65 (0.37–1.14), *p* = 0.1287) and CERENAL (6.5 months versus 4.8 months; HR_CERENAL 0–3_ = 0.69 (0.47–2.56), *p* = 0.1800) and, whereas such an effect was not observed for RTOG RPA; Figure S2D–F).

## 4. Discussion

Based on the increasing use of targeted therapies to treat BMs, we explored the prognostic potential of CERENAL next to the validated and widely used RTOG RPA and DS-GPA in a heterogeneous population of patients with BMs receiving systemic therapy following local brain therapy. CERENAL is a novel prognostic score introduced in previous research from our group. El Ali et al. [21] reported that CERENAL is an independent prognosticator for brain-metastasized RCC patients treated with targeted agents. Extrapolation to a larger, more heterogeneous cohort resulted in an ad hoc adaptation of several of the incorporated prognostic factors towards the final CERENAL score. Firstly, the previous cut-off value for age (≤50 years old) was considered too low, as only 17% of patients developed BMs before the age of 50. Based on the median age at primary diagnosis of 70, 64, and 62 years old for lung cancer, RCC, and breast cancer, respectively [23,24,25], the cut-off for age as a prognostic factor was increased to ≤60 years old. Next, a novel cut-off (≥3) was implemented for number of BM lesions, as the previous cut-off value (≥2) no longer showed prognostic value. This lack in significance can be explained by the fact that SRS or stereotactic boost after WBRT is only administered in patients with few BMs and as an association existed in our cohort between number of BMs and receiving SRS or stereotactic boost. Lastly, receiving a stereotactic boost after WBRT or undergoing neurosurgery can be attributed with the same improved survival outcome. In this respect, the original prognostic index, namely, receiving SRS, was modified to receiving SRS, stereotactic boost after WBRT, or undergoing neurosurgery.

In the current study, primary local therapy administered was significantly associated with the stratification among brain prognostic scores, a logical finding, as the factors used in the prognostic scores are indicated in various guidelines with respect to which local therapy to administer in patients suffering from BMs [6,8]. It was interesting to see that the updated CERENAL model was associated with RTOG RPA with only a concordance of 58%, despite comparable prognostic parameters included in each prognostic score. An important difference was seen in the classification of patients in the good/intermediate prognosis RTOG RPA class (I/II). Off these patients, 48.0% were classified as CERENAL 0–3, whereas the other 52.0% had a poor prognosis according to the CERENAL score. A larger discordance was observed between RTOG RPA and DS-GPA. Although nearly all patients with a DS-GPA of ≤2.0 were classified as RTOG RPA III (96%), only 48 out of 150 patients with a RTOG RPA I/II were categorized as DS-GPA >2.0. DS-GPA and CERENAL, on the other hand, illustrated good agreement, with 75% of patients being correctly classified between poor and favorable prognostic scores. The discordance between RTOG RPA and the other prognostic scores is most likely attributable to more prognostic parameters, including disease-specific covariates, included in the CERENAL score and DS-GPA. As a result, these discordances probably explain the differences observed in survival outcomes per prognostic scores in our study.

Overall, the median OS was 6.3 months, with 2-year and 5-year OS of 13% and 6%, respectively. These survival data concur with historical data, although these numbers are lower compared to OS outcomes of more recent studies reporting median OS outcomes between eight and seventeen months [26,27,28,29]. This could be explained by the fact that our population was treated in the period 2006 to 2017, whereas the recent increase in OS is attributed to more recent advances in both local and systemic therapeutic approaches [27,30]. As only 93 patients received subsequent systemic therapy, of whom only 62% received a type of targeted/immunotherapy, the absence of novel therapeutics could explain the poor prognosis in our population. In addition, only 7% of patients in our cohort were included in the favorable RTOG RPA class I, whereas RTOG RPA class III was overrepresented in our study (25%). It has been reported that the distribution of patients in prognostic RTOG RPA classes I, II, and III is approximately 20%, 65%, and 15%, respectively [22,27]. The imbalance in stratification among RTOG RPA, together with the frailty of our patients—more than half presented with three or more BMs at diagnosis—accounts for the observed reduced survival outcome.

The current treatment paradigm for BMs relies on a number of clinical factors, such as tumor(s) size/total volume, mass effect, lesion number, and patient performance status. Several prognostic scores focusing on general health parameters, e.g., RTOG RPA, have been developed in order to enable the prognostication of patients with BMs and to aid in clinical decision-making [6,8,31]. Compared to recent research using RTOG RPA, our OS and DSS outcomes are comparable between class I, II, and III [32]. Notably in our study, patients classified as RTOG RPA I had a shorter median OS in comparison to RTOG RPA II patients. This may again be due to an imbalance in RTOG RPA classification groups. OS according to the CERENAL score was also lower than the outcome observed in the original study in which CERENAL was evaluated [21]. This can logically be ascribed to the more heterogeneous cohort with poorer survival outcome in comparison to the initial RCC cohort. Otherwise, in the current era of personalized medicine, the use of general health parameters can be considered less appropriate, which implies a need to incorporate more disease-specific covariates. Over the past decade, the group of Sperduto et al. has developed DS-GPA for numerous solid malignancies [16,17,18,19,20]. The OS data in our real-world cohort is in accordance with the known literature, except for DS-GPA 3.5–4.0. In our cohort, this subgroup only had a median OS of 6.4 months compared to the 17 to 52 months reported in the literature. The low OS outcome in our study is mostly likely attributable to the fact that only six patients had a favorable DS-GPA score of 3.5–4.0, thus again illustrating the frailty in our patient cohort.

Univariate analysis highlighted RTOG RPA, DS-GPA, and CERENAL as OS prognosticators in the ITT cohort. In addition, CERENAL showed high prognostic potential in both patient cohorts stratified according to subsequent systemic therapy following BM diagnosis. As a consequence, CERENAL was withheld as an independent OS prognosticator in all multivariate analyses (ITT cohort, cohort with subsequent systemic therapy, and cohort without subsequent systemic therapy). Otherwise, the univariate OS outcome for RTOG-RPA proved to be more strongly associated in patients receiving subsequent systemic therapy, whereas for DS-GPA, the opposite association was noticed. Due to these similarities, only RTOG RPA proved to be an independent prognosticator for OS following multivariate analysis, except in the patient cohorts receiving no subsequent systemic therapy. Interestingly, DS-GPA was not withheld in any of the multivariate models for OS.

For DSS, a similar survival outcome to OS was observed. However, multivariate analysis only identified the CERENAL prognostic score as an independent prognosticator for DSS, irrespective of administration of subsequent systemic therapy. This further highlights the potential of the CERENAL score to be used as prognostic model in patients with BMs. It remains, however, curious that DS-GPA, which is a validated and routinely used brain prognostic score, did not prove to have independent prognostic value in our real-world cohort. One potential explanation for the lack of significance may be the fact that the time of BM diagnosis in our patient cohort ranged from 2006 to 2017. Therefore, these patients may have received less targeted therapy, thus having less benefit from a prognostic favorable covariate.

Lastly, PFS was significantly different between low and high DS-GPA scores. This finding would indicate that the incorporation of more disease-specific and treatment-related covariates brings prognostic properties with regard to suffering from disease progression. Nevertheless, responding well to systemic treatment following BM diagnosis does not necessarily correlate to having an improved OS or DSS outcome, as indicated previously. Next, for CERENAL also, a nearly significant association with PFS was found. This association is probably due to the use of both general prognostic parameters as well as BM therapy-related parameters in the CERENAL model. Simultaneously, incorporating disease-specific variables (DS-GPA) and BM therapy-related parameters (CERENAL) can explain why RTOG-RPA was unable to differentiate between poor and favorable PFS, as RTOG RPA incorporates only three basic general health parameters (age, KPS, and other extracranial metastases).

With regard to patient outcome, it is clear that implementing a brain prognostic score, which only incorporates general health parameters, does not suffice and that there is an urgent need for more complex nomograms that incorporate both general patient status as well as disease-specific parameters, such as genomic alterations, grading, etc. Recently, more primary site-related prognostic grading systems have been developed due to the hypothesis that BMs behave differently depending on the primary tumor [5]. Here, we have shown that more favorable DS-GPA scores are indeed associated with prolonged PFS, although DS-GPA had no impact on OS or DSS outcome. Therefore, it appears to be imperative to not only focus on disease-specific variables, such as mutational status, but also to take BM therapy-related parameters into account. In this regard, our proposed CERENAL proves to be a good brain metastatic prognostic score for a heterogeneous population, enabling the differentiation of patients from low-ranging OS to a more favorable outcome. As CERENAL seems unaffected by the primary tumor type, our findings indicated that brain prognostic scores composed of general health-related prognostic factors are still usable and feasible in patients with BMs. Moreover, the CERENAL score can be considered more applicable versus RTOG-RPA, as the radiotherapeutic treatment regimen is also incorporated into the CERENAL score, thus taking the extent of the BMs and received local therapy into account. Nevertheless, it remains to be evaluated if the prognostic potential of the CERENAL nomogram can be improved by the incorporation of other disease-specific parameters, as is the case for DS-GPA. Such an evolution in nomogram adaptation is crucial, as we have noticed a long tail in the OS and DSS outcomes for CERENAL and for DS-GPA, accounting for ten to fifteen percent of patients. Due to the continuous use of novel therapies, that also target BMs, it is to be expected that the number of long-term survivors will increase. It is therefore of vital importance to identify patients who will have long-term survival following BM diagnosis, as was stipulated by Hügel et al. [29].

Due to the retrospective design, the absence of data is one of the major limitations in our study. Several data (e.g., diameter of lesions) were not available in the patient records, thus making us unable to compare our CERENAL score to more novel prognostic scores or parameters nowadays included into the BM treatment paradigm [33,34,35]. In addition, genetic screening data from most of these patients were scanty, which hampered the inclusion of the potential prognostic effects of presence/absence of druggable mutations in our CERENAL model. This limitation can also account for the fact that some of the patients may have been misclassified in DS-GPA, therefore potentially resulting in a non-significant outcome on multivariate analysis. Next, as patients were treated in the period 2006–2017, we were unable to examine the effect of novel therapies, such as immune-checkpoint inhibitors and novel class targeted therapies. This limitation, combined with the heterogeneity of our patient population, may reduce the statistical inference with regard to the prognostic abilities of the CERENAL score. Lastly, an extensive validation of CERENAL and its separate prognostic factors is missing, which currently inhibits CERENAL from being used in a more routine clinical setting. In this regard, the prognostic effect of neurosurgery versus SRS only versus boost after WBRT is unclear. The hypothesis in our study is that having underwent either one of these local therapies serves the same purpose, namely, the treatment of brain oligometastatic disease with the objective of maximizing survival. It therefore remains to be determined if grouping these local therapies together results in any potential bias.

Nevertheless, the results of the current study encourage future studies for the continuous evaluation of the CERENAL score. Extensive prospective validation is advised in patients treated with novel therapies, such as immune-checkpoint inhibitors and next-generation small molecules, as it has been reported that these therapeutics can have a significant clinical impact on BMs [28,36,37,38,39]. In this way, the true prognostic potential of CERENAL can be assessed. In addition, further improvement of the CERENAL model is needed by performing additional subanalysis into the tumor histopathology and mutational status of each tumor indication, as was done for DS-GPA [40]. Such a modification holds particular value in the current era of personalized medicine.

## 5. Conclusions

In the current retrospective study, we evaluated the prognostic value of the CERENAL score in brain-metastasized cancer patients. The main objective was to determine the prognostic outcome of CERENAL in patients who did or did not receive subsequent systemic therapy. CERENAL enables to identify patients with favorable OS and DSS outcomes, irrespective of administration of subsequent systemic therapy following primary local therapy for their BMs. Future studies are encouraged for the continuous evaluation of the CERENAL score. Finally, further improvement of the model is warranted in the current era of personalized medicine, e.g., by implementing tumor histopathology and mutational status.

## Figures and Tables

**Table 1:** Calculation of brain metastatic prognostic scores.

RTOG RPA ^§^	I		II			III		
Age at diagnosis of first BM	<65		all			all		
	and		and			and		
KPS at diagnosis of first BM	≥70		≥70			<70		
	and		and			and		
Other extracranial metastases	No		No/Yes			No/Yes		
DS-GPA ^#^								
Breast carcinoma	0	0.5		1			1.5	2
Age	≥60	<60						
KPS	≤60	70–80		90–100				
Number of brain metastatic lesions	>1	1						
Subtype	ER− PR− HER2−	ER+ PR+ HER2−					HER2+	
Gastrointestinal carcinoma	0	0.5		1			1.5	2
Age	≥60	<60						
KPS	<80			80				90–100
Extracranial metastases present	Present	Absent						
Number of brain metastatic lesions	>3	2–3		1				
RCC	0	0.5		1			1.5	2
KPS	<80			80				90–100
Extracranial metastases present	Present	Absent						
Number of brain metastatic lesions	>3	2–3		1				
Hemoglobin (g/dL)	≤11	11.1–12.5		>12.5				
Melanoma	0	0.5		1			1.5	2
Age	≥70	<70						
KPS	≤70	80		90–100				
Extracranial metastases present	Present			Absent				
Number of brain metastatic lesions	>4	2–4		1				
*BRAF* mutation	Absent	Present						
NSCLC—adenocarcinoma	0	0.5		1			1.5	2
Age	≥70	<70						
KPS	≤70	80		90–100				
Extracranial metastases present	Present			Absent				
Number of brain metastatic lesions	≥5	1–4						
*EGFR* and *ALK* mutation	Absent	Present						
PD-L1 status (%)	<1	≥1						
NSCLC—non-adenocarcinoma	0	0.5		1			1.5	2
Age	≥70	<70						
KPS	≤60			70			80	90–100
Extracranial metastases present	Present			Absent				
Number of brain metastatic lesions	≥5	1–4						
DS-GPA ^#^								
SCLC	0	0.5		1			1.5	2
Age	≥75	<75						
KPS	≤60	70		80			90	100
Extracranial metastases present	Present	Absent						
Number of brain metastatic lesions	≥8	4–7		1–3				
CERENAL ^#^	0				1			
Age at diagnosis of first BM *	≤60 years				>60 years			
KPS at diagnosis of first BM	>70				≤70			
Other extracranial metastases	No				Yes			
PD at diagnosis of first BM	No				Yes			
Number of brain metastatic lesions *	1–2				≥3			
Received NS, SRS or boost to WBRT *	Yes				No			

§ Patients are subdivided into classes according to the combination of all three criteria. # Final score is obtained by sum of all individual points. * Adaptation of cut-offs in comparison to previously reported CERENAL score [21] based on ad hoc calculations in the current larger dataset: age at diagnosis of first brain metastasis > 60 years old instead of >50 years old; number of brain metastatic lesions ≥ 3 instead of ≥2; received neurosurgery, SRS, or boost to WBRT instead of received SRS. ALK, anaplastic lymphoma kinase; BM, brain metastasis; DS-GPA, disease-specific graded prognostic assessment; EGFR, epidermal growth factor receptor; ER, estrogen receptor; HER2, human epidermal growth factor receptor 2; KPS, Karnofsky performance status; NS, neurosurgery; NSCLC, non-small-cell lung cancer; PD, progressive disease; PD-L1, programmed cell death ligand 1; PR, progesterone receptor; RCC, renal cell carcinoma; RTOG RPA, Radiation Therapy Oncology Group recursive partitioning analysis; SCLC, small-cell lung cancer; SRS, stereotactic radiosurgery; WBRT, whole-brain radiotherapy.

**Table 2:** Association of brain metastasis prognostic scores.

A. With each other brain metastasis prognostic score								
CERENAL		0–3			4–6			*p*
RTOG RPA	I/II	72			78			<0.0001
	III	6			44			
		ρ = 0.459; *p* < 0.0001						
CERENAL		0–3			4–6			*p*
DS-GPA	0.0–2.0	39			111			<0.0001
	2.5–4.0	39			11			
		ρ = −0.681; *p* < 0.0001						
RTOG RPA		I/II			III			*p*
DS-GPA	0.0–2.0	102			48			<0.0001
	2.5–4.0	48			2			
		ρ = −0.480; *p* < 0.0001						
B. With administered primary local therapy for BMs								
Primary local therapy		No	WBRT 20 Gy	WBRT 30 Gy		WBRT 30 Gy + NS/SRS/boost	SRS	*p*
RTOG RPA	I/II	2	78	8		58	4	<0.0001
	III	5	39	2		4	0	
		ρ = −0.348; *p* < 0.0001						
DS-GPA	0.0–2.0	6	101	6		35	2	0.0002
	2.5–4.0	1	16	4		27	2	
		ρ = 0.437; *p* < 0.0001						
CERENAL	0–3	3	14	4		54	3	<0.0001
	4–6	4	103	6		8	1	
		ρ = −0.638; *p* < 0.0001						
C. With subsequent systemic therapy								
Subsequent systemic therapy		No			Yes			*p*
RTOG RPA	I/II	71			79			0.0025
	III	36			14			
		ρ = −0.143; *p* = 0.0440						
DS-GPA	0.0–2.0	80			70			0.9349
	2.5–4.0	27			23			
		ρ = 0.056; *p* = 0.4338						
CERENAL	0–3	39			39			0.4286
	4–6	68			54			
		ρ = −0.023; *p* = 0.7519						

Significance was calculated using Fisher’s exact test. ρ and the respective *p* value were determined using rank correlation testing. BMs, brain metastases; DS-GPA, diagnosis-specific graded prognostic assessment; Gy, gray; NS, neurosurgery; RTOG RPA, Radiation Therapy Oncology Group recursive partitioning analysis; SRS, stereotactic radiosurgery; WBRT, whole-brain radiotherapy.

**Table 3:** OS analysis for prognostic scores.

Parameter			*N*	Univariate Analysis			Multivariate Analysis	
				Median OS (95% CI)	HR (95% CI)	*p*	HR (95% CI)	*p*
A. ITT	Subsequent θ	Yes	93	10.4 (8.9–13.1)	1			
		No	107	3.9 (2.9–4.5)	2.10 (1.55–2.86)	<0.0001		
	RTOG RPA	I/II	150	8.1 (6.4–9.8)	1		1	
		III	50	2.9 (2.0–4.3)	2.19 (1.47–3.27)	0.0001	1.46 (1.03–2.07)	0.0330
	DS-GPA	0.0–2.0	150	5.7 (4.3–6.9)	1		1	
		2.5–4.0	50	9.4 (6.4–18.5)	0.56 (0.41–0.77)	0.0003	0.80 (0.53–1.21)	0.2934
	CERENAL	0–3	78	10.9 (7.3–16.6)	1		1	
		4–6	122	4.5 (3.5–5.9)	2.28 (1.69–3.09)	<0.0001	2.08 (1.50–2.88)	<0.0001
B. No θ	RTOG RPA	I/II	71	4.5 (3.9–5.8)	1		1	
		III	36	2.1 (1.5–2.9)	1.67 (1.05–2.65)	0.0319	1.20 (0.77–1.88)	0.4209
	DS-GPA	0.0–2.0	80	3.0 (2.5–4.0)	1		1	
		2.5–4.0	27	5.9 (4.1–15.8)	0.49 (0.32–0.75)	0.0010	0.75 (0.41–1.37)	0.3511
	CERENAL	0–3	39	5.8 (4.5–14.1)	1		1	
		4–6	68	2.6 (2.3–3.0)	2.49 (1.64–3.77)	<0.0001	2.51 (1.62–3.89)	<0.0001
C. θ	RTOG RPA	I/II	79	11.6 (9.4–13.3)	1		1	
		III	14	5.6 (3.5–8.9)	4.80 (2.03–11.3)	0.0003	1.97 (1.06–3.66)	0.0330
	DS-GPA	0.0–2.0	70	9.9 (7.8–12.9)	1		1	
		2.5–4.0	23	16.6 (8.8–31.9)	0.57 (0.36–0.90)	0.0148	0.79 (0.44–1.41)	0.4236
	CERENAL	0–3	39	14.4 (9.4–29.2)	1		1	
		4–6	54	8.9 (6.5–10.9)	2.38 (1.52–3.74)	0.0002	2.06 (1.27–3.34)	0.0032

Median time is given in months. θ, systemic therapy; CI, confidence interval; DS-GPA, diagnosis-specific graded prognostic assessment; HR, hazard ratio; ITT, intention to treat; N, number; OS, overall survival; RTOG RPA, Radiation Therapy Oncology Group recursive partitioning analysis.

**Table 4:** DSS analysis for prognostic scores.

Parameter			*N*	Univariate Analysis			Multivariate Analysis	
				Median DSS (95% CI)	HR (95% CI)	*p*	HR (95% CI)	*p*
A. ITT	Subsequent θ	Yes	88	10.9 (9.1–13.1)	1			
		No	104	3.9 (2.9–4.5)	2.16 (1.58–2.97)	<0.0001		
	RTOG RPA	I/II	145	8.1 (6.4–10.4)	1		1	
		III	47	2.9 (2.0–4.5)	2.09 (1.38–3.16)	0.0005	1.39 (0.97–2.00)	0.0732
	DS-GPA	0.0–2.0	143	5.8 (4.5–7.1)	1		1	
		2.5–4.0	49	9.4 (6.4–18.7)	0.55 (0.40–0.76)	0.0003	0.80 (0.52–1.23)	0.3093
	CERENAL	0–3	76	10.7 (7.3–16.6)	1		1	
		4–6	116	4.5 (3.5–6.1)	2.32 (1.70–3.16)	<0.0001	2.32 (1.69–3.20)	<0.0001
B. No θ	RTOG RPA	I/II	69	4.5 (3.5–5.8)	1		1	
		III	35	2.3 (1.5–2.9)	1.58 (0.98–2.54)	0.0589	1.15 (0.73–1.81)	0.5435
	DS-GPA	0.0–2.0	77	3.0 (2.5–3.9)	1		1	
		2.5–4.0	27	5.9 (4.1–15.8)	0.48 (0.31–0.73)	0.0008	0.74 (0.39–1.39)	0.3493
	CERENAL	0–3	37	5.8 (4.5–15.8)	1		1	
		4–6	67	2.6 (2.3–3.5)	2.50 (1.64–3.82)	<0.0001	2.56 (1.63–4.02)	<0.0001
C. θ	RTOG RPA	I/II	76	11.6 (9.4–13.5)	1		1	
		III	12	6.4 (3.5–11.5)	4.34 (1.75–10.8)	0.0015	1.83 (0.94–3.56)	0.0744
	DS-GPA	0.0–2.0	66	10.4 (8.4–13.1)	1		1	
		2.5–4.0	22	16.6 (7.1–37.5)	0.55 (0.35–0.89)	0.0140	0.74 (0.40–1.36)	0.3306
	CERENAL	0–3	39	14.4 (9.4–29.2)	1		1	
		4–6	49	9.4 (6.9–11.6)	2.31 (1.45–3.68)	0.0004	2.26 (1.42–3.60)	0.0006

Median time is given in months. θ, systemic therapy; CI, confidence interval; DS-GPA, diagnosis-specific graded prognostic assessment; HR, hazard ratio; ITT, intention to treat; N, number; RTOG RPA, Radiation Therapy Oncology Group recursive partitioning analysis.

## Data Availability

The data presented in this study are available on request from the corresponding author (not publicly available due to European General Data Protection Regulation).

## Supplementary Materials

The following supporting information can be downloaded at https://www.mdpi.com/article/10.3390/curroncol32020074/s1. Figure S1: STARD diagram; Figure S2: PFS outcome (first subsequent systemic therapy) for brain metastasis prognostic scores; Table S1: Clinical characteristics in the intention-to-treat cohort (*N* = 200); Table S2: Clinical characteristics at brain metastasis diagnosis in ITT cohort (*N* = 200); Table S3: Survival outcomes for separate brain prognostic scores.
