# Supplementary material for: Prognostication of Brain-Metastasized Patients Receiving Subsequent Systemic Therapy: A Single-Center Long-Term Follow-Up
Source: Curr Oncol. 2025 Jan 28;32(2):74. doi: 10.3390/curroncol32020074 (PMC11853900; doi:10.3390/curroncol32020074)
Supplement: Supplementary file 1 [file curroncol-32-00074-s001.zip › curroncol-3385787-supplementary.pdf]

Current Oncology

MDPI

*Supplementary Materials*

# Prognostication of Brain-Metastasized Patients Receiving Subsequent Systemic Therapy: A Single-Center Long-Term Follow-Up

## 1. Supplementary figures

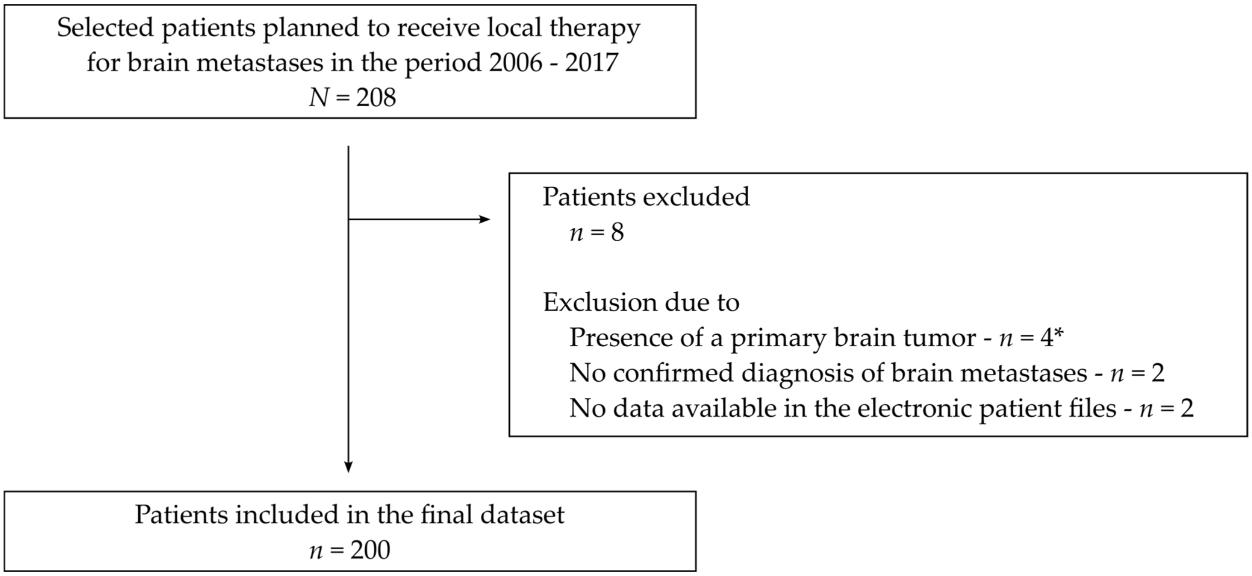


Figure S1. STARD diagram

* Included two glioblastoma, one astrocytoma and one ependymoma

 https://doi.org/10.3390/curroncol32020074

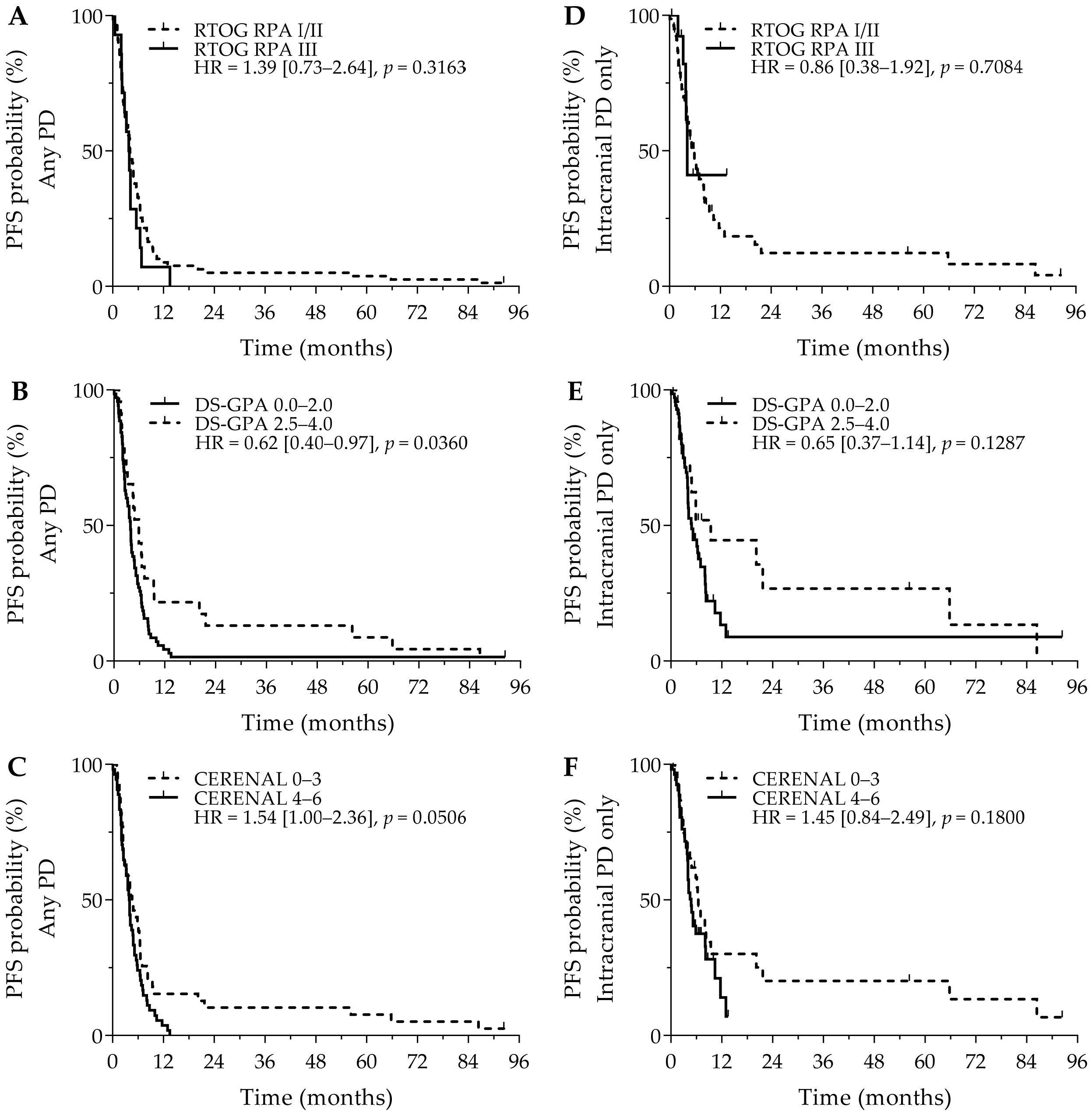


Figure S2. PFS outcome (first subsequent systemic therapy) for brain metastasis prognostic scores. Y-axis depicts cumulative survival (%), X-axis depicts survival time in months. PFS survival curves are demonstrated for: (A) RTOG RPA in the ITT cohort ($p = 0.3163$), (B) CERENAL in the ITT cohort ($p = 0.0506$), (C) DS-GPA in the ITT cohort ($p = 0.0360$), (D) RTOG RPA with intracranial PD as end point ($p = 0.7084$), (E) CERENAL with intracranial PD as end point ($p = 0.1800$), and (F) DS-GPA with intracranial PD as end point ($p = 0.1287$). HR [95 CI] are indicated in each survival graph. CI, confidence interval; DS-GPA: disease-specific graded prognostic assessment; HR, hazard ratio; ITT, intention-to-treat; PD, progressive disease; PFS, progression-free survival; RTOG RPA, Radiation Therapy Oncology Group Recursive partitioning analysis.

## 2. Supplementary tables

Table S1. Clinical characteristics in the intention-to-treat cohort (*n* = 200)

| | | |
|---|---|---|
| Gender | Female | 106 (53.0) |
| | Male | 94 (47.0) |
| Age at diagnosis of primary tumor (years) | | 59 (20-80) |
| Type of primary tumor | Breast | 31 (15.5) |
| | Gastro-intestinal | 18 (9.0) |
| | Genitourinary # | 34 (17.0) |
| | Gynecological | 7 (3.5) |
| | Head and neck | 4 (2.0) |
| | NSCLC – adenocarcinoma | 55 (27.5) |
| | NSCLC – non-adenocarcinoma | 10 (5.0) |
| | SCLC | 11 (5.5) |
| | Melanoma | 27 (13.5) |
| | Bone | 1 (0.5) |
| | CUP | 2 (1.0) |
| Surgery of primary tumor | No | 93 (46.5) |
| | Radical | 85 (42.5) |
| | Cytoreductive | 17 (8.5) |
| | Unknown | 5 (2.5) |
| Primary tumor stage | T1 | 36 (18.0) |
| | T2 | 51 (25.5) |
| | T3 | 50 (25.0) |
| | T4 | 33 (16.5) |
| | Unknown | 30 (15.0) |
| Primary nodal stage | N0 | 56 (28.0) |
| | N1 | 41 (20.5) |
| | N2+ | 75 (37.5) |
| | Unknown | 28 (14.0) |
| Presence of M+ disease at diagnosis of primary tumor | No | 92 (46.0) |
| | Yes | 108 (54.0) |

All data are *N* (%), except for Age diagnosis primary tumor: median (range). # Genitourinary cancers are all renal cell carcinoma except for one prostate carcinoma. CUP, cancer of unknown primary; M+, metastatic; N, nodal; NSCLC, non-small cell lung cancer; SCLC, small cell lung cancer; T, tumor.

Table S2. Clinical characteristics at BM diagnosis in ITT cohort ($n$ = 200)

| | | | |
|---|---|---|---|
| Age at diagnosis of BM (years) | | 62 | (21-81) |
| Time from diagnosis of primary tumor to BM (months) | | 15 | (0-267) |
| Brain metastasis at diagnosis of primary tumor | No | 146 | (73.0) |
| | Yes | 54 | (27.0) |
| Other metastatic sites involved at BM diagnosis | No sites | 31 | (15.5) |
| | Lungs | 97 | (48.5) |
| | Lymph nodes | 89 | (44.5) |
| | Liver | 64 | (32.0) |
| | Bones | 77 | (38.5) |
| | Adrenal gland | 42 | (21.0) |
| | Other | 65 | (32.5) |
| PD at diagnosis of first BM (primary tumor included) | No | 71 | (35.5) |
| | Yes | 129 | (64.5) |
| KPS | < 50 | 3 | (1.5) |
| | 50 | 14 | (7.0) |
| | 60 | 33 | (16.5) |
| | 70 | 43 | (21.5) |
| | 80 | 65 | (32.5) |
| | 90 | 42 | (21.0) |
| Localization of BM | Supratentorial | 99 | (49.5) |
| | Infratentorial | 23 | (11.5) |
| | Both | 78 | (39.0) |
| Number of BMs | 1 | 61 | (30.5) |
| | 2 | 34 | (17.0) |
| | 3 | 16 | (8.0) |
| | 4 | 28 | (14.0) |
| | 5 | 15 | (7.5) |
| | 6 | 10 | (5.0) |
| | 7 | 5 | (2.5) |
| | 8+ | 31 | (15.5) |
| Neurological symptoms | No | 44 | (22.0) |
| | Yes | 156 | (78.0) |
| Hemoglobin (g/dL) | ($n$ = 178) | 12.6 | (7.7-17) |
| Neutrophil count (x$10^3$/μL) | ($n$ = 143) | 5640 | (570-20088) |
| Lymphocyte count (x$10^3$/μL) | ($n$ = 143) | 1220 | (250-4000) |
| Calcium (mmol/L) | ($n$ = 170) | 2.34 | (1.82-2.72) |
| Alkaline phosphatase (U/L) | ($n$ = 174) | 82 | (25-652) |
| Lactate dehydrogenase (U/L) | ($n$ = 160) | 223.5 | (49-4290) |
| C-reactive protein (mg/L) | ($n$ = 173) | 8.2 | (0-281.2) |
| Primary local therapy | NS + WBRT 10x3Gy or 12x2.5Gy | 21 | (10.5) |
| | NS + WBRT 10x3Gy or 12x2.5Gy + boost | 2 | (1.0) |
| | NS + WBRT 5x4Gy | 3 | (1.5) |
| | NS + SRS | 1 | (0.5) |
| | WBRT 10x3Gy or 12x2.5Gy | 10 | (5.0) |
| | WBRT 10x3Gy or 12x2.5Gy + boost | 36 | (18.0) |
| | WBRT 5x4Gy | 117 | (58.5) |
| | SRS | 3 | (1.5) |
| | No | 7 | (3.5) |

Table S2. Clinical characteristics at BM diagnosis in ITT cohort ($n$ =200) – continued

| | | |
|---|---|---|
| Received systemic therapy following diagnosis of BM | No | 107 (53.5) |
| | First-line | 50 (25.0) |
| | Second-line | 23 (11.5) |
| | Third-line | 11 (2.5) |
| | Fourth-line | 6 (3.0) |
| | Fifth-line | 1 (0.5) |
| | Sixth-line | 2 (1.0) |
| RTOG RPA | I | 14 (7.0) |
| | II | 136 (68.0) |
| | III | 50 (25.0) |
| DS-GPA | 0.0–1.0 | 69 (34.5) |
| | 1.5–2.0 | 81 (40.5) |
| | 2.5–3.0 | 44 (22.0) |
| | 3.5–4.0 | 6 (3.0) |
| CERENAL | 0 | 2 (1.0) |
| | 1 | 18 (9.0) |
| | 2 | 29 (14.5) |
| | 3 | 29 (14.5) |
| | 4 | 62 (31.0) |
| | 5 | 42 (21.0) |
| | 6 | 18 (9.0) |

All data are *N* (%), except for Age at diagnosis of BM, Time from diagnosis of primary tumor to BM; Hemoglobin, Neutrophil count, Lymphocyte count, Calcium, Alkaline phosphatase, Lactate dehydrogenase, C-reactive protein: median (range). BM, brain metastasis; DS-GPA, disease-specific graded prognostic assessment; Gy, gray; ITT, intention-to-treat; NS, neurosurgery; PD, progressive disease; RTOG RPA, Radiation Therapy Oncology Group Recursive partitioning analysis; SRS, stereotactic radiosurgery; WBRT, whole-brain radiotherapy.

Table S3. Survival outcomes for separate brain prognostic scores

| A) PFS | | | | | | | | | |
|---|---|---|---|---|---|---|---|---|---|
| Group | ITT | | | Subsequent therapy | | | No subsequent therapy | | |
| Score | *n* | Median PFS (95% CI) | *p* | *n* | Median PFS (95% CI) | *p* | *n* | Median PFS (95% CI) | *p* |
| Overall | | | | 93 | 4.0 (3.2–4.9) | – | | | |
| RTOG RPA | | | | | | | | | |
| I | | | | 4 | 1.6 (1.2–4.4) | 0.0141 | | | |
| II | | | | 75 | 4.5 (3.3–5.6) | | | | |
| III | | | | 14 | 3.8 (2.1–5.6) | | | | |
| DS-GPA | | | | | | | | | |
| 0.0–1.0 | | | | 13 | 2.6 (2.0–3.8) | 0.0047 | | | |
| 1.5–2.0 | | | | 39 | 4.4 (3.6–6.1) | | | | |
| 2.5–3.0 | | | | 20 | 4.9 (2.5–9.4) | | | | |
| 3.5–4.0 | | | | 3 | 5.9 (1.6–65.8) | | | | |
| CERENAL | | | | | | | | | |
| 0–1 | | | | 5 | 5.6 (1.6–5.6) | 0.0250 | | | |
| 2 | | | | 15 | 3.9 (1.9–6.5) | | | | |
| 3 | | | | 17 | 5.9 (2.1–8.3) | | | | |
| 4 | | | | 30 | 4.9 (3.3–6.8) | | | | |
| 5 | | | | 20 | 3.6 (2.1–5.2) | | | | |
| 6 | | | | 6 | 2.8 (0.1–4.8) | | | | |

| B) OS | | | | | | | | | |
|---|---|---|---|---|---|---|---|---|---|
| Group | ITT | | | Subsequent therapy | | | No subsequent therapy | | |
| Score | *n* | Median OS (95% CI) | *p* | *n* | Median OS (95% CI) | *p* | *n* | Median OS (95% CI) | *p* |
| Overall | 200 | 6.3 (5.4–8.0) | – | 93 | 10.4 (8.9–13.1) | – | 107 | 3.9 (2.9–4.5) | – |
| RTOG RPA | | | | | | | | | |
| I | 14 | 6.4 (4.0–30.6) | 0.0002 | 4 | 6.4 (3.2–30.6) | 0.0015 | 10 | 5.9 (1.0–66.1) | 0.0109 |
| II | 136 | 8.1 (6.4–9.8) | | 75 | 11.6 (9.4–13.5) | | 61 | 4.2 (3.4–5.4) | |
| III | 50 | 2.9 (2.0–4.3) | | 14 | 5.6 (3.5–8.9) | | 36 | 2.1 (1.5–2.9) | |
| DS-GPA | | | | | | | | | |
| 0.0–1.0 | 69 | 4.3 (2.9–5.7) | 0.0001 | 31 | 6.4 (4.5–8.9) | 0.0007 | 38 | 2.5 (1.6–4.1) | 0.0077 |
| 1.5–2.0 | 81 | 8.0 (5.0–10.4) | | 36 | 12.9 (9.9–13.5) | | 42 | 3.4 (2.9–5.0) | |
| 2.5–3.0 | 44 | 9.4 (6.4–18.7) | | 18 | 9.6 (7.1–36.5) | | 24 | 5.9 (4.0–18.5) | |
| 3.5–4.0 | 6 | 6.4 (5.4–70.0) | | 3 | 30.6 (6.4–70.0) | | 3 | 5.8 (5.4–15.8) | |
| CERENAL | | | | | | | | | |
| 0–1 | 18 | 15.8 (5.4–66.1) | <0.0001 | 5 | 90.1 (13.1–90.1) | <0.0001 | 13 | 6.5 (5.3–40.4) | 0.0005 |
| 2 | 31 | 8.4 (4.9–18.7) | | 15 | 9.5 (5.4–22.6) | | 16 | 5.4 (3.4–20.1) | |
| 3 | 27 | 10.9 (6.2–18.5) | | 17 | 16.6 (7.3–31.6) | | 10 | 5.8 (1.0–11.7) | |
| 4 | 64 | 6.1 (3.9–8.8) | | 30 | 11.6 (8.1–13.5) | | 34 | 2.9 (2.4–3.9) | |
| 5 | 42 | 4.3 (2.2–5.7) | | 20 | 6.9 (4.3–10.9) | | 22 | 1.9 (1.1–3.0) | |
| 6 | 18 | 3.5 (1.8–6.2) | | 6 | 3.9 (1.1–13.1) | | 12 | 2.5 (1.6–6.3) | |

Table S3. Survival outcomes for separate brain prognostic scores – continued

| C) DSS | | | | | | | | | | | | |
|---|---|---|---|---|---|---|---|---|---|---|---|---|
| Group | ITT | | | Subsequent therapy | | | No subsequent therapy | | |
| Score | *n* | Median DSS (95% CI) | *p* | *n* | Median DSS (95% CI) | *p* | *n* | Median DSS (95% CI) | *p* |
| Overall | 192 | 6.4 (5.4–8.1) | – | 88 | 10.9 (9.1–13.1) | – | 104 | 3.9 (2.9–4.5) | – |
| RTOG RPA | | | | | | | | | |
| I | 13 | 10.4 (4.0–66.1) | 0.0007 | 3 | 10.4 (6.4–30.6) | 0.0074 | 10 | 5.9 (1.0–66.1) | 0.0212 |
| II | 132 | 8.1 (6.4–10.4) | | 73 | 12.9 (9.4–13.5) | | 59 | 4.1 (3.0–5.4) | |
| III | 47 | 2.9 (2.0–4.5) | | 12 | 6.4 (3.5–11.5) | | 35 | 2.3 (1.5–2.9) | |
| DS-GPA | | | | | | | | | |
| 0.0–1.0 | 66 | 4.5 (2.9–5.9) | 0.0001 | 29 | 6.5 (4.5–9.8) | 0.0007 | 37 | 2.5 (1.8–4.1) | 0.0072 |
| 1.5–2.0 | 77 | 8.1 (5.0–10.9) | | 37 | 13.1 (10.4–14.4) | | 40 | 3.0 (2.6–4.5) | |
| 2.5–3.0 | 43 | 9.4 (6.4–18.7) | | 19 | 16.6 (7.1–37.5) | | 24 | 5.9 (4.0–18.5) | |
| 3.5–4.0 | 6 | 6.4 (5.4–70.0) | | 3 | 30.6 (6.4–70.0) | | 3 | 5.8 (5.4–15.8) | |
| CERENAL | | | | | | | | | |
| 0–1 | 17 | 15.8 (5.8–90.1) | <0.0001 | 5 | 90.1 (13.1–90.1) | 0.0001 | 12 | 14.1 (5.4–66.1) | 0.0004 |
| 2 | 31 | 8.4 (4.9–18.7) | | 15 | 9.5 (5.4–22.6) | | 16 | 5.4 (3.4–20.1) | |
| 3 | 26 | 10.9 (6.2–21.3) | | 17 | 16.6 (7.3–31.6) | | 9 | 5.8 (1.0–11.7) | |
| 4 | 61 | 6.1 (3.9–8.8) | | 27 | 13.1 (8.8–14.4) | | 34 | 2.9 (2.4–3.9) | |
| 5 | 41 | 4.3 (2.2–5.7) | | 19 | 7.0 (4.3–10.9) | | 22 | 1.9 (1.1–3.0) | |
| 6 | 16 | 3.5 (1.8–6.3) | | 5 | 6.2 (1.1–13.1) | | 11 | 3.5 (1.6–6.3) | |

Median survival times are given in months. CI, confidence interval; DS-GPA, disease-specific graded prognostic assessment; DSS, disease-specific survival; ITT, intention-to-treat; OS, overall survival; PFS, progression-free survival; RTOG RPA, Radiation Therapy Oncology Group Recursive partitioning analysis; SRS, stereotactic radiosurgery; WBRT, whole-brain radiotherapy.
